# Development and evaluation of a self-administered on-line test of memory and attention for middle-aged and older adults

**DOI:** 10.3389/fnagi.2014.00335

**Published:** 2014-12-10

**Authors:** Angela K. Troyer, Gillian Rowe, Kelly J. Murphy, Brian Levine, Larry Leach, Lynn Hasher

**Affiliations:** ^1^Neuropsychology and Cognitive Health Program, Baycrest Centre for Geriatric CareToronto, ON, Canada; ^2^Department of Psychology, University of TorontoToronto, ON, Canada; ^3^Rotman Research Institute, Baycrest Centre for Geriatric CareToronto, ON, Canada

**Keywords:** memory, executive attention, cognitive assessment, internet-based assessment, psychometric evaluation

## Abstract

There is a need for rapid and reliable Internet-based screening tools for cognitive assessment in middle-aged and older adults. We report the psychometric properties of an on-line tool designed to screen for cognitive deficits that require further investigation. The tool is composed of measures of memory and executive attention processes known to be sensitive to brain changes associated with aging and with cognitive disorders that become more prevalent with age. Measures included a Spatial Working Memory task, Stroop Interference task, Face-Name Association task, and Number-Letter Alternation task. Normative data were collected from 361 healthy adults age 50–79 who scored in the normal range on a standardized measure of general cognitive ability. Participants took the 20-minute on-line test on their home computers, and a subset of 288 participants repeated the test 1 week later. Analyses of the individual tasks indicated adequate internal consistency, construct validity, test-retest reliability, and alternate version reliability. As expected, scores were correlated with age. The four tasks loaded on the same principle component. Demographically-corrected z-scores from the individual tasks were combined to create an overall score, which showed good reliability and classification consistency. These results indicate the tool may be useful for identifying middle-aged and older adults with lower than expected scores who may benefit from clinical evaluation of their cognition by a health care professional.

## Introduction

Life expectancy is increasing globally (World Health Organization, 2014), creating an older adult population that is rapidly growing. Because age is the strongest risk factor for cognitive decline, the need for cognitive screening is likely to rise proportionately. With increased access to computers and the Internet, particularly among older adults (Wagner et al., 2010), interactive web-based cognitive assessment that identifies individuals in need of further evaluation has become more feasible and has the potential to be extremely useful. With this in mind, we created and evaluated a self-administered on-line cognitive screening tool for middle-aged and older adults.

Our tool joins an emerging suite of digital and on-line tests. Some of these tools are well-validated, but require trained professionals to administer and/or score the tests (e.g., Weintraub et al., 2013; Scharre et al., 2014) or consist primarily of informant reports of cognitive decline (Brandt et al., 2013). Other tests have been used in very large numbers to evaluate cognitive training gains (e.g., www.lumosity.com; www.cambridgebrainsciences.com), but do not yet have published studies supporting the psychometric properties of the tests themselves. To our knowledge, few self-administered computerized tests of cognitive ability have undergone peer-reviewed psychometric evaluation. One recent test (Zakzanis and Azarbehi, 2014) includes five measures that, when combined into a composite score, show a correlation with age, distinguish normal from clinical groups, and have excellent overall reliability; it is not clear that the various tasks load on a single or multiple factors, however, and statistics are not provided for the individual subtests. Another test (Lee et al., 2012) includes 13 measures that correlate with age and load onto two separate factors of processing speed and memory; reliability data are not provided for the composite scores or individual subtests.

Our goal was to create a psychometrically valid, reliable and easy-to-use self-assessment tool that can be used by individuals to determine whether or not they should raise their concerns about memory with their primary care provider. As such, our focus was on establishing the range of normal performance on measures of cognitive abilities known to recruit brain regions affected by aging and by early cognitive disorders.

Region specific brain changes in normal aging predominately affect the prefrontal cortex and medial temporal regions including the hippocampus (Raz, 2000; Raz and Rodrigue, 2006), with neuropathological aging typically associated with even greater changes in medial temporal structures (Head et al., 2005; Dickerson et al., 2009). Both of these brain regions play an important role in higher level cognitive processes. The prefrontal cortex supports strategic aspects of memory and attention, including working memory—or holding information ‘in-mind’ to guide decisions and actions—and executive attention, such as interference control and cognitive flexibility (Kane and Engle, 2002). The hippocampus supports memory processes such as binding information together to form an accurate representation (reviewed in Rosenbaum et al., 2012).

Consistent with these age-related brain changes, it is well known that episodic and associative memory, working memory, and executive attention decline in normal cognitive aging (reviewed in Hasher and Zacks, 1988; Luo and Craik, 2008; Old and Naveh-Benjamin, 2008; Salthouse, 2010). Some of these same cognitive changes are also seen in early cognitive disorders (Bäckman et al., 2005; Troyer et al., 2008; Johns et al., 2012).

Based on these age-related changes in the brain and cognition, we selected four tasks of memory and executive attention and modified them to accommodate on-line self-administration:

1. A spatial working memory task (Passingham, 1985; Owen et al., 1990; Duff and Hampson, 2001) was selected that requires participants to efficiently locate multiple pairs of hidden shapes in an array and avoid erroneously returning to previously searched locations. Brain lesion and functional neuroimaging studies have confirmed the essential role of the prefrontal cortex in this type of task (Jonides et al., 1993; Courtney et al., 1998).
2. A Stroop task (Stroop, 1935) was used to examine attentional control and processing speed. To accommodate responding by key press, a counting variant of the task (e.g., Bush et al., 2006) was developed in which participants identified the number of words shown on each trial. During interference trials, the number of words was incongruent with the meaning of the word (e.g., the word “three” was written two times). Both the standard and counting variants of the Stroop task show greater interference effects in older relative to younger adults (reviewed in Salthouse and Meinz, 1995; Davidson et al., 2003)—due to either age-related slowing (Verhaeghen and De Meersman, 1998) or reduced inhibitory control (Hasher and Zacks, 1988)—and are sensitive to dementia (Girelli et al., 2001; Hutchison et al., 2010) and frontal lobe damage (Stuss et al., 2001b).
3. A face-name association task was chosen because associative memory is dependent on the integrity of the hippocampus (Mayes et al., 2004) and because the task is sensitive to both normal aging and mild cognitive impairment (Troyer et al., 2011, 2012). Because changes in hippocampal volume occur early in pathological aging including Alzheimer’s disease (Head et al., 2005), this measure may be particularly sensitive for distinguishing normal memory changes from those of a more serious nature.
4. The final task was a variation of Trail Making Test part B (Army Individual Test Battery, 1944; reviewed in Tombaugh, 2004). On this task, participants alternate sequencing numbers and letters in ascending order as quickly and accurately as possible. This task is multifactorial (Sánchez-Cubillo et al., 2009), engaging working memory to maintain the current sequence while searching for the next number or letter, flexibility to alternate attention between the two sequences, and processing speed. Older adults show greater difficulty on these tasks compared to younger adults, due to both age-related decline in processing speed as well as age differences in executive cognitive processes (Salthouse et al., 2000). The frontal lobes significantly, although not exclusively, support the cognitive operations involved in this task (Stuss et al., 2001a; Zakzanis et al., 2005).

Overall, the goals of this research were to: (a) assess the feasibility of our web-based platform for test administration; (b) assess the reliability and construct validity of the measures; and (c) obtain normative data that could be used to assist older adults in evaluating their subjective memory concerns. Because the measures were based on well-established cognitive tests, it was expected they would exhibit good internal consistency, construct validity, and reliability. We expected the tasks to be inter-correlated and, given the selection of two memory tasks and two tasks of executive attention that they would load on two separate factors.

## Materials and methods

The study protocol was approved by the Research Ethics Board at Baycrest Centre for Geriatric Care.

### Participants

Adults age 50 and older were recruited via advertisements and from participant and market-research databases. For evaluating psychometric test properties, we included data from all 396 participants who completed the test on at least one occasion and who did not produce extreme outliers on testing. For calculating normative data, we excluded 35 participants with a self-reported history of medical conditions known to affect cognition (e.g., traumatic brain injury, stroke, mild cognitive impairment, current depression) and/or those scoring below the normal range on a cognitive screening test (i.e., less than 31 on the modified Telephone Interview for Cognitive Status; Welsh et al., 1993).

We recruited participants with demographic characteristics—including age, sex, and educational attainment—to create a normative sample that was representative of the North American population (United States Census Bureau, 2011; Statistics Canada, 2012a,b). Demographic data for our sample are presented in Table 1.

**Table 1:** **Sample demographics**.

	5-year age groups						
	50–54 (*n* = 39)	55–59 (*n* = 72)	60–64 (*n* = 82)	65–69 (*n* = 57)	70–74 (*n* = 54)	75–79 (*n* = 57)	All (*n* = 361)
Age (mean, SD)	52 (1.3)	57 (1.4)	62 (1.3)	67 (1.2)	72 (1.2)	77 (1.8)	65 (8.2)
Sex (*n*, %):
Females	24 (62)	39 (54)	47 (57)	31 (54)	27 (50)	34 (60)	202 (56)
Males	15 (38)	33 (46)	35 (43)	26 (46)	27 (50)	23 (40)	159 (44)
Education (*n*, %):
Less than high school	4 (10)	5 (7)	5 (6)	12 (21)	7 (13)	8 (14)	41 (11)
High school	8 (20)	19 (26)	29 (35)	16 (28)	12 (22)	16 (28)	100 (28)
University	18 (46)	33 (46)	32 (39)	15 (26)	16 (30)	23 (40)	137 (38)
Post-graduate degree	9 (23)	15 (21)	16 (20)	14 (25)	19 (35)	10 (18)	83 (23)

*Note. Education is the highest level of education completed*.

Most participants received no monetary compensation. Because of difficulty recruiting individuals with less than a high school education, near the end of the recruitment period we offered $75 to improve recruitment in this group. Subsequent analyses indicated that paid (*n* = 9) and unpaid (*n* = 32) participants with less than high school education did not differ on the four targeted test scores, *F*_(4,36)_ < 1, *p* = 0.57, $\eta_{p}^{2}$ = 0.08.

### Tasks

We selected and developed computerized tasks based on existing clinical and experimental tasks known to be sensitive to subtle cognitive changes associated with aging and age-related cognitive disorders. In designing and selecting the tasks, we sought to keep the total duration of the battery at around 20 min.

We conducted pilot testing with 140 participants over 3 iterations of test development. The first iteration involved testing participants in the laboratory under our direct observation to ensure that they understood task instructions and responded appropriately. The remaining iterations involved participants taking the test from their own homes. After each iteration, we adjusted tasks as needed to ensure that response properties and distributions were appropriate.

The final tasks were programed in ASP.NET, JavaScript, and Adobe Flash, and the program was hosted on the Microsoft Azure cloud computing platform. Tasks could be completed from PC or Macintosh desk-top and laptop computers, but not from tablet computers or mobile devices. Completing the tasks required users to have an Internet connection, a recent version of an Internet browser (i.e., Internet Explorer 7 or above, Safari version 4 or above, Firefox version 10 or above, and Google Chrome any version), and a recent version of Adobe Flash Player (version 10 or above).

Tasks were administered in a fixed order: Spatial Working Memory, Stroop Interference, Face-Name Association, and Letter-Number Alternation. Administration of each task was preceded by detailed instructions showing sample task stimuli. The Stroop interference and letter-number alternation tasks also had practice trials during which feedback was provided for incorrect responses. On these practice trials, errors were immediately identified, and participants were required to make a correct response before proceeding to the next item.

Four versions of each task were developed using different task stimuli (for the Spatial Working Memory and Face-Name Association tasks), different spatial locations (for the Spatial Working Memory and Letter-Number Alternation tasks), and different orders of test stimuli (for the Stroop Interference task).

**Figure 1:**
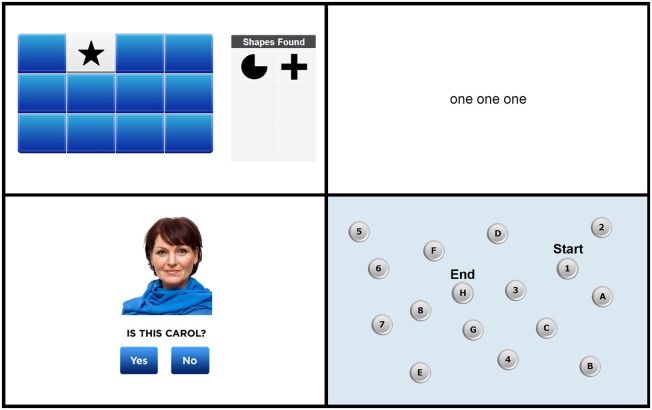
**Screen shots of the Spatial Working Memory task, Stroop Interference task, Face-Name Association task, and Letter-Number Alternation task, respectively**. Adapted from www.cogniciti.com with permission from Cogniciti Inc.

Screen shots from each of the four tasks are shown in Figure 1. The full test battery is available from www.cogniciti.com.

#### Spatial working memory

Our task was based on procedures developed by Duff and Hampson (2001). A 4 by 3 array of rectangular tiles was displayed on the computer screen. The array contained 6 pairs of shapes (e.g., triangles, pentagons, circles, or sunbursts), with each tile hiding one shape. Participants clicked with the mouse on tiles to reveal the shape beneath. Only two shapes could be seen at any time, and after each pair of clicks, both shapes were shown for 1 s. Each time two matching shapes were uncovered, that shape appeared in a “shapes found” box located to the right of the target array. Thus, participants did not have to remember which shape pairs they had already located, rather they had to keep track of previously searched locations within working memory to reduce errors (e.g., uncovering two unmatched locations or two previously matched locations). The participant’s task was to find all 6 pairs of shapes in as few clicks as possible. Once all pairs had been discovered, additional trials, using the identical array, were administered immediately and again at the end of the end of the entire test session. The number of responses and the time in seconds required to find all 6 pairs of shapes were recorded for each of the three trials.

#### Stroop interference

Based on the original task developed by Stroop (1935), we created a number-word interference task using simple words (e.g., “call” and “then”) and written number words (i.e., “one,” “two,” and “three”). On each trial, participants were required to indicate the number of words shown on the screen by pressing the number keys 1, 2, or 3 as quickly as possible without making any mistakes. Three types of trials were presented in an inter-mixed, pseudo-random order: neutral trials, consisting of non-number words (e.g., “and and and”); congruent trials, in which the number words corresponded to the number of words presented (e.g., “two two”); and incongruent trials, in which the number words did not correspond to the number of words presented (e.g., “three”). There were 30 trials of each condition, for a total of 90 trials. Participants were not given feedback on their responses and were not allowed to correct any incorrect responses. This task was self-paced, with each stimulus remaining on the screen until the participant responded (for a maximum of 4 s), and a 500 ms inter-stimulus interval between trials. Any failures to respond within 4 s were scored as incorrect responses, and these occurred very rarely (i.e., 0.1% of all responses). Accuracy for each response and reaction times (RTs) for correct responses were recorded and were averaged for each of the three trial types.

#### Face-name association

This task was adapted from our previous work (Troyer et al., 2012). Male and female faces reflecting a wide range of ages and ethnic groups were taken from on-line databases (e.g., Shutterstock, iStock, Veer). First names were taken from a listing of the most common baby names from the past 100 years (Social Security Administration, 2013) and were paired with age- and gender-appropriate faces. A total of 24 face-name pairs were presented individually for 3 s each (with a 500 ms inter-stimulus interval) across two presentation trials. Immediately following the second list presentation, a yes/no recognition test consisting of 12 intact and 12 recombined face-name pairs was administered. Participants were instructed to click on a “yes” button for face-name combinations included in the encoding list and a “no” button for recombined items. This recognition task was self-paced, with each face-name pair remaining on the screen until the participant responded (for a maximum of 10 s), and a 500 ms inter-stimulus interval between trials. Any failures to respond within 10 s were scored as incorrect responses, and these occurred rarely (i.e., 0.5% of all responses). Accuracy for each response and RTs for correct responses were recorded.

#### Letter-number alternation

Our task was based on the standard trail-making test (Army Individual Test Battery, 1944) used in neuropsychological assessment. A display of 16 buttons, each containing a number from 1 to 8 or a letter from A to H, was shown on the screen. Participants were instructed to click on the numbers and letters in alternating order (i.e., 1, A, 2, B, 3, and so on), starting with the number 1 and ending with the letter H, as quickly and as accurately as possible. With each click, a line appeared connecting the consecutive items. Incorrect responses were immediately identified, and participants were required to determine and click on the correct number or letter before proceeding. Accuracy and total time required to complete the sequence were measured.

### Procedures

Participants provided consent and completed a medical history and cognitive screen by telephone. Subsequently, they received two e-mail messages 1 week apart containing instructions and links for completing the on-line test in their own homes. The on-line component consisted of reading general instructions for the test, completing a demographic and health questionnaire, completing the four tasks, and providing (optional) feedback about the research.

A total of 396 participants completed the test at least once, 288 of whom completed it on both occasions. Subsequent analyses indicated that participants taking the test only once (*n* = 108) and those taking it twice (*n* = 288) did not differ on the four targeted test scores, *F*_(4,391)_ < 1, *p* = 0.43, $\eta_{p}^{2}$ = 0.01. Of those completing the test twice, participants received either the same (*n* = 76) or an alternate (*n* = 212) version on the second occasion. The four test versions were counterbalanced across test occasions and were used approximately equally often.

Of the 797 occasions on which the test was started during our recruitment period, there were 696 (87%) completions. Of these, 656 (94%) test completions produced data within 3 standard deviations of the group mean on each of the four tasks and were not considered to be outliers.

## Results

Descriptive data obtained from participants’ first test occasion, collapsed across the four test versions, are presented in Table 2. All analyses described subsequently were conducted on raw test scores, with the exception of those involving the overall score, which is derived from demographically corrected normative scores.

**Table 2:** **Descriptive test data for scores obtained on the first test occasion**.

	5-year age groups					
	50–54 (*n* = 39)	55–59 (*n* = 72)	60–64 (*n* = 82)	65–69 (*n* = 57)	70–74 (*n* = 54)	75–79 (*n* = 57)
**Spatial Working Memory:**						
Trial 1 responses	36.4 (12.6)	41.0 (19.1)	42.6 (18.9)	43.4 (23.9)	42.1 (16.2)	45.3 (17.1)
Trial 2 responses	29.6 (13.7)	31.2 (17.3)	31.7 (14.3)	32.0 (12.6)	34.2 (12.7)	38.7 (15.2)
Trial 3 responses	26.7 (10.8)	30.6 (15.6)	30.0 (13.7)	29.2 (12.7)	31.6 (19.6)	33.9 (12.3)
*Trial 1–3 responses	92.8 (25.0)	102.8 (37.3)	104.3 (39.1)	104.5 (36.7)	107.9 (34.2)	117.9 (30.2)
Trial 1 time to completion (s)	82 (35)	98 (45)	108 (65)	114 (61)	116 (56)	126 (63)
Trial 2 time to completion (s)	69 (37)	76 (40)	76 (40)	83 (40)	90 (37)	101 (56)
Trial 3 time to completion (s)	56 (26)	66 (32)	68 (34)	68 (32)	78 (43)	82 (37)
**Stroop Interference:**						
Congruent: % accuracy	98 (6)	99 (3)	100 (2)	96 (14)	99 (16)	100 (1)
Neutral: % accuracy	98 (5)	99 (4)	99 (2)	96 (16)	99 (2)	99 (3)
Incongruent: % accuracy	96 (5)	96 (5)	97 (4)	95 (14)	98 (4)	96 (5)
Congruent: median RT (ms)	931 (154)	969 (180)	1038 (169)	1086 (167)	1075 (172)	1107 (162)
Neutral: median RT (ms)	957 (158)	993 (179)	1052 (152)	1092 (159)	1100 (155)	1132 (160)
*Incongruent: median RT (ms)	1027 (174)	1058 (204)	1129 (173)	1159 (171)	1163 (176)	1210 (178)
**Face-Name Association:**						
Hits (out of 12)	10.6 (1.3)	10.2 (1.9)	9.7 (1.7)	10.2 (1.7)	9.8 (1.8)	9.4 (1.7)
False alarms (out of 12)	2.1 (2.5)	2.1 (1.7)	2.8 (1.9)	2.5 (1.9)	2.4 (1.5)	3.2 (2.3)
*% accuracy	85 (12)	84 (11)	79 (13)	82 (10)	80 (11)	75 (13)
vMedian RT (ms)	2023 (494)	2385 (847)	2427 (802)	2524 (610)	2766 (942)	2938 (1081)
**Letter-Number Alternation:**						
% accuracy	95 (10)	95 (11)	96 (8)	94 (11)	96 (9)	94 (13)
*Time to completion (s)	31 (13)	35 (18)	32 (13)	35 (13)	34 (15)	38 (17)

*Note. RT = reaction time for correct responses. Data are presented as means (or medians) and standard deviations*.

*Target variable for each respective task.

### Determination of normative scores

One target measure for each task was selected based on an examination of the distribution of scores as well as analyses of internal consistency and reliability. These target measures are indicated with asterisks in Table 2, and include the number of responses required to complete each trial summed across the three trials of the Spatial Working Memory task, median RT on correct responses to incongruent trials on the Stroop Interference task, overall percent accuracy on the 24 test trials of the Face-Name Association task, and time required to complete the sequence on the Letter-Number Alternation task.

For each task, z-scores were calculated from the normative sample. To determine which characteristics to take into account in calculating the normative z-scores, we used MANOVAs and repeated-measures ANOVA to examine the effects of demographic and test variables on the four test scores. There were significant overall effects of age group, *F*_(20,1420)_ = 3.59, *p* < 0.001, $\eta_{p}^{2}$ = 0.05, education group, *F*_(12,1068)_ = 1.79, *p* = 0.045, $\eta_{p}^{2}$ = 0.02, test version, *F*_(12,1068)_ = 2.46, *p* < 0.004, $\eta_{p}^{2}$ = 0.03, and test occasion, *F*_(4,261)_ = 16.27, *p* < 0.001, $\eta_{p}^{2}$ = 0.20. There was no significant effect of sex on overall performance, *F*_(4,365)_ = 1.07, *p* = 0.37, $\eta_{p}^{2}$ = 0.01. For those characteristics with significant overall effects, we examined the effect sizes for each individual task. Based on these analyses, normative data were broken down by age group for the Spatial Working Memory and Letter-Number Alternation tasks, by age group and test version for the Face-Name Association task, and by age group and test occasion for the Stroop Interference task. An overall score was calculated as the mean of the four z scores, and a cut-off score of −1.50 was determined based on observed clusters of scores at the low end of the distribution curve. Eight of the 361 participants in the normative sample obtained a score below this cut-off, yielding a failure rate of 2%.

### Reliability

As a measure of internal consistency, the split-half correlation of the 24 responses on the Face-Name Association test was calculated as 0.62. Cronbach’s alpha for the 30 incongruent items of the Stroop Interference task was 0.96. The other two tasks did not have a sufficient number of trials to calculate internal consistency.

Test-retest reliability was calculated from the 76 participants who completed the same test version on two occasions. As seen in Table 3, test-retest reliability ranged from *r*_(74)_ = 0.49 to 0.82 for the individual tasks, and was 0.72 for the overall score. All correlations were significant, *p*’s < 0.01.

**Table 3:** **Test-retest and alternate-version reliability**.

	Test-retest (*n* = 76)	Alternate-version (*n* = 212)
Spatial Working Memory	0.49	0.52
Stroop Interference	0.83	0.82
Face-Name Association	0.66	0.48
Letter-Number Alternation	0.49	0.52
Overall score	0.72	0.69

*Note. Target values are the number of responses to completion summed across the three trials of the Spatial Working Memory task, median RT on correct responses to incongruent trials on the Stroop Interference task, overall percent accuracy on the Face-Name Association task, and time to completion on the Letter-Number Alternation task; overall score is the mean of the four demographically corrected z scores. Values presented are Pearson’s r. All correlations are significant at *p* < 0.01*.

Alternate-version reliability was calculated from the 212 participants who completed different versions of the test on two occasions. As seen in Table 3, alternate-version reliabilities ranged from *r*_(210)_ = 0.48 to 0.82 for the individual tasks, and was 0.69 for the overall score. All correlations were significant, *p*’s < 0.01.

### Validity

As a measure of construct validity, correlations between age and the target measures for each task were calculated. As seen in Table 4, these correlations were small to medium in size, *r*_(394)_ = −0.20 to 0.31, and were all statistically significant, *p*’s < 0.01.

**Table 4:** **Correlations with age and between tasks**.

	Spatial Working Memory	Stroop Interference	Face-Name Association	Letter-Number Alternation
Age	0.17	0.31	−0.20	0.14
Spatial Working	1
Memory
Stroop Interference	0.18	1
Face-Name	−0.27	−0.18	1	
Association
Letter-Number	0.21	0.30	−0.22	1
Alternation

*Note: Target values are the number of responses to completion summed across the three trials of the Spatial Working Memory task, median RT on correct responses to incongruent trials on the Stroop Interference task, overall percent accuracy on the Face-Name Association task, and time to completion on the Letter-Number Alternation task. Values presented are Pearson’s r. *N* = 396. All correlations are significant at *p* < 0.01*.

We further assessed construct validity of the Spatial Working Memory task by examining learning over repeated trials. Consistent with expectations, performance on the three trials differed significantly in the number of responses required for completion, *F*_(2,734)_ = 76.7, *p* < 0.001, $\eta_{p}^{2}$ = 0.17, and the amount of time taken, *F*_(2,732)_ = 118.2, *p* < 0.001, $\eta_{p}^{2}$ = 0.24. Examination of the data in Table 2 showed the expected performance improvements across the three learning trials.

We assessed the construct validity of the Stroop Interference task by examining the effects of congruency. Consistent with the well-known Stroop effect, performance on the three types of Stroop trials differed significantly in both accuracy, *F*_(2,734)_ = 72.8, *p* < 0.001, $\eta_{p}^{2}$ = 0.17, and median RT for correct responses, *F*_(2,734)_ = 391.5, *p* < 0.001, $\eta_{p}^{2}$ = 0.52. Examination of the data in Table 2 shows that, numerically, accuracy scores decreased and speed scores increased from congruent to neutral to incongruent trials.

As a measure of convergent validity, we examined inter-task correlations of the target measures, which are shown in Table 3. These correlations were small to medium in size, *r*_(394)_ = −0.27 to 0.30, and were statistically significant, *p*’s < 0.01.

To determine the component structure, we conducted an initial principle components analysis (PCA) from the first test occasion (*n* = 396). This showed that all 4 tasks loaded on a single component (Eigenvalue = 1.61), with individual component loadings ranging from 0.58 to 0.75. Given our inclusion of two types of cognitive tasks—namely, memory and speeded executive attention tasks—we conducted another PCA with the same data, forcing two components and using a varimax rotation. The Spatial Working Memory task and Face-Name Association task loaded highly on the first component (Eigenvalue = 1.61), with rotated component loadings of 0.75 and 0.80, respectively. This was interpreted as a memory component. The Stroop Interference and Letter-Number Alternation tasks loaded highly on the second component (Eigenvalue = 0.95), with rotated component loadings of 0.86 and 0.71, respectively. This was interpreted as a speeded executive attention component.

To replicate the component structure, we repeated these PCAs on the subsample (*n* = 288) that took the test on a second occasion. The results were similar to the first analyses, with all 4 tasks loading on a single component (Eigenvalue = 1.61) and individual component loadings ranging from 0.57 to 0.79. When forcing two components and using a varimax rotation, the Spatial Working Memory task and Face-Name Association task loaded highly (0.73 and 0.80, respectively) on the first component (Eigenvalue = 1.61), and the Stroop Interference and Letter-Number Alternation tasks loaded highly (0.89 and 0.65, respectively) on the second component (Eigenvalue = 0.98).

### Classification consistency of overall test score

The standard error of measurement at the cut-off score of −1.50 was 0.35 (95% confidence interval = −1.51 to −0.56). Classification consistency, measured as percent of participants who scored above or below the cut-off on both test occasions, was excellent, 98%, Fisher’s exact *p* < 0.001. Most participants (273 out of 282) obtained scores above the cut-off at both occasions, and 3 participants obtained scores below the cut-off at both occasions. The 6 participants who obtained a score below the cut-off on only one occasion also obtained low scores on the remaining occasion, ranging from −1.14 to −0.64.

## Discussion

We validated an on-line cognitive screening instrument to provide rapid, reliable information regarding relative preservation or impairment in cognition relative to one’s age peers. Rather than assessing gross mental status, as is the case in standard dementia screening tools, we focused on specific cognitive abilities that may precede the onset of a full-blown dementia syndrome. Thus the goal of this study was to define the normal range of responses in a healthy sample to determine appropriate cut-off scores that may signal the need for more in-depth assessment. We drew from clinical neuropsychological assessment and cognitive neuroscience research on healthy aging and dementia to provide measures with the greatest potential for identifying the changes in memory and executive functioning that herald atypical brain aging.

Our results suggest that a web-based cognitive assessment can feasibly provide meaningful results for individual test takers. Technical and human errors were minimized, such that 87% of tests started were fully completed. Of the tests that were completed, 94% produced results within the expected range on all 4 tasks, suggesting that there were no undue errors that introduced bias into the results.

These feasibility findings are notable, given the challenges of automated, remote testing. Whereas such instruments can never be as flexible as in-person evaluation, extensive piloting insured that respondents could follow the instructions and produce data of sufficient quality. We also utilized a web-based platform that could collect data in a consistent manner across a variety of browsers and hardware configurations, and we created extensive instructions, practice trials, and feedback to anticipate any potential problem in comprehension of instructions or task execution. In this respect, our web-based administration mimicked the guidance provided by one-on-one testing.

Detailed psychometric testing showed acceptable reliability of our test. The test-retest reliability of 0.72 for the overall test score provides evidence for stability over time. Although test-retest reliabilities for some of the individual tasks were relatively lower, this is not an unusual finding. In fact, our tasks compare favorably with those of standard neuropsychological tests measuring similar constructs administered to middle- and older-adult age groups. That is, reliability coefficients for our Letter-Number Alternation (*r* = 0.49) and Stroop Interference task (*r* = 0.83) are the same as or higher than those from the Trail-Making Test switching condition (*r* = 0.55) and the Color-Word Interference Test inhibition condition (*r* = 0.50) from the Delis-Kaplan Executive Function System (Delis et al., 2001). Similarly, the reliabilities of our Spatial Working Memory (*r* = 0.49) and Face-Name Association (*r* = 0.66) tasks are similar to those of the immediate and delayed Designs Spatial task (*r*’s = 0.56 and 0.50) and immediate Face Recognition (*r* = 0.64) from the Wechsler Memory Scale (Wechsler, 1997, 2009). Additionally, alternate form reliability for the overall test score (*r* = 0.69) supported the use of this tool for serial testing, where practice effects could artificially elevate scores if the same form were used. Notably, given the difference in reliabilities for the overall score vs. the individual tasks, the main score for interpretation is the overall score.

Construct validity was supported by modest but reliable correlations between test performance and age, as expected given age-related changes in speed, attention, memory, and executive functioning (reviewed in Salthouse, 2010). Moreover, within-test comparisons across conditions were consistent with established psychological principles. That is, the expected learning curve was demonstrated across trials of the Spatial Working Memory task and the expected interference effect was demonstrated on the Stroop Interference task.

The principal components analysis conservatively identified a single factor solution that was used to derive cut-off scores for this measure. This cut-off identified eight out of 361 (2%) participants as candidates for further assessment. There was also evidence in support of a two-factor solution that reflected constructs of memory and executive attention in the context of speeded responding. The possibility of a one- or two-factor solution is not surprising given recent theoretical work suggesting that attention regulation underlies memory (Hasher et al., 1999; Healey et al., 2014). Further research is required to establish the validity of the factor structure with respect to gold-standard measures. If supported, a two-factor solution could provide more nuanced feedback relating to selective preservation or impairment in mnemonic or executive processes.

In spite of the limitations in web-based cognitive assessment, we attained a high degree of control over the delivery of instructions and automated management of responses, as demonstrated by our feasibility, reliability, and validity data. It is nonetheless acknowledged that individuals who complete on-line testing do so in an uncontrolled environment where fatigue, medications, mood, time of day, effort and numerous other factors might affect test performance. Whereas these same factors also affect performance in a standard testing situation, the examiner can take these into account when interpreting the data. For these reasons, in general, we recommend that a detailed history be included with web-based assessments so that endorsement of potentially confounding factors can be reported and subsequently taken into consideration. Similarly, feedback delivered to the participant should contain caveats about the limitations of web-based testing. The inclusion of validated alternate forms allows the option of repeat testing in the case of ambiguous results or transient factors affecting test performance. Although web-based testing will always be less controlled than in-person testing, we note that many individuals may not seek in-person assessment due to anxiety, lack of access, or other factors. In this respect, web-based testing provides useful feedback to guide individuals in making a decision whether to pursue further assessment.

As our sample was limited to adults aged 50–79, this instrument is not recommended for individuals falling outside of this age range. We had difficulty recruiting unpaid volunteers with lower education, so we paid a small number of volunteers to fill these cells. Although we could detect no statistically significant effect of payment on test results, we nonetheless recommend caution in interpreting scores from those with lower education, which can affect performance for reasons other than cognitive decline. The availability of the test to the public will result in larger sample sizes that will allow us to examine more closely the impact of specific demographic variables on our task.

It is expected that those with advanced cognitive decline would fall below the observed cut-off scores, but this study did not include individuals with mild cognitive impairment, early Alzheimer’s disease, or other age-related conditions. Our goal was to specify a cut-off score as an empirical criterion to identify those falling outside the normal range of cognitive functioning for follow-up assessment, not to diagnose brain disease. We are currently assessing the sensitivity and specificity of this instrument in relation to brain disease. It is clear, however, that this or any other stand-alone test cannot be used for diagnosis outside of a clinical setting where individual differences, medical findings, and other relevant factors would be considered.

Future research will also be needed to demonstrate concurrent validity of our tasks. Specifically, it will be important to understand how our on-line tasks compare to tasks accepted to measure similar cognitive constructs. This would provide evidence of the ability of our test to measure working memory, associative memory, and executive attention.

Overall, our findings support the feasibility, reliability, and validity of this online assessment tool and its use as a screening measure to detect greater than expected changes in cognitive functioning in middle-aged and older adults. The need for such a test is likely to grow, as the projected number of adults in this age group increases, along with the incidence of age-related cognitive disorders such as dementia. The standard paradigm of one-on-one assessment in a doctor’s office cannot support this increasing need, which will be composed of both those with genuine cognitive decline due to incipient dementia and the “worried well” seeking reassurance. On-line assessment that does not require individualized attention from a healthcare professional has the potential to significantly reduce demand on the healthcare system, allowing resources to be more efficiently targeted to those truly in need.

## Author statement

Authorship is listed in order of contribution.

## Conflict of interest statement

Drs. Troyer, Rowe, Murphy, and Levine report consultation fees from Cogniciti. The authors also report grants from the Federal Economic Development Agency for Southern Ontario and from Cogniciti.

## References

[B1] Army Individual Test Battery (1944). Manual of Directions and Scoring. Washington, DC: War Department, Adjutant General’s Office.

[B2] Bäckman L. Jones S. Berger A.-K. Jonsson Laukka E. Small B. J. (2005). Cognitive impairment in preclinical Alzheimer’s disease: a meta-analysis. Neuropsychology 19, 520–531. 10.1037/0894-4105.19.4.520 16060827

[B3] Brandt J. Sullivan C. Burrell L. E. Rogerson M. Anderson A. (2013). Internet-based screening for dementia risk. PLOS One 8:e57476. 10.1371/journal.pone.0057476 23437393 PMC3578821

[B4] Bush G. Whalen P. J. Shin L. M. Rauch S. L. (2006). The counting stroop: a cognitive interference task. Nat. Protoc. 1, 230–233. 10.1038/nprot.2006.35 17406237

[B5] Courtney S. M. Petit L. Maisog J. M. Ungerleider L. G. Haxby J. V. (1998). An area specialized for spatial working memory in human frontal cortex. Science 279, 1347–1351. 10.1126/science.279.5355.1347 9478894

[B6] Davidson D. J. Zacks R. T. Williams C. C. (2003). Stroop interference, practice and aging. Neuropsychol. Dev. Cogn. B Aging Neuropsychol. Cogn. 10, 85–98. 10.1076/anec.10.2.85.14463 17203134 PMC1761647

[B7] Delis D. C. Kaplan E. Kramer J. H. (2001). Delis-Kaplan Executive Function System Technical Manual. San Antonio: Pearson.

[B8] Dickerson B. C. Feczko E. Augustinak J. C. Pacheco J. Morris J. C. Fischl B.. (2009). Differential effects of aging and Alzheimer’s disease on medial temporal lobe cortical thickness and surface area. Neurobiol. Aging 30, 432–440. 10.1016/j.neurobiolaging.2007.07.022 17869384 PMC3703585

[B9] Duff S. J. Hampson E. (2001). A sex difference on a novel spatial working memory task in humans. Brain Cogn. 47, 470–493. 10.1006/brcg.2001.1326 11748902

[B10] Girelli L. Sandrini M. Cappa S. Butterworth B. (2001). Number-stroop performance in normal aging and Alzheimer’s-type dementia. Brain Cogn. 46, 144–149. 10.1016/s0278-2626(01)80053-1 11527315

[B11] Hasher L. Zacks R. T. (1988). “Working memory, comprehension and aging: a review and a new view,” in The Psychology of Learning and Motivation, ed Bower G. H. (San Diego, CA: Academic Press), 193–225.

[B12] Hasher L. Zacks R. T. May C. P. (1999). “Inhibitory control, circadian arousal and age,” in Attention and Performance, XVII, Cognitive Regulation of Performance: Interaction of Theory and Application, eds Gopher D. Koriat A. (Cambridge, MA: MIT Press), 653–675.

[B13] Head D. Snyder A. Z. Girton L. E. Morris J. C. Buckner R. L. (2005). Frontal-hippocampal double dissociation between normal aging and Alzheimer’s disease. Cereb. Cortex 15, 732–739. 10.1093/cercor/bhh174 15371293

[B14] Healey M. K. Ngo K. W. J. Hasher L. (2014). Below baseline suppression of competitors during interference resolution by younger but not older adults. Psychol. Sci. 25, 145–151. 10.1177/0956797613501169 24214245

[B15] Hutchison K. A. Balota D. A. Duchek J. M. (2010). The utility of Stroop task switching as a marker for early-stage Alzheimer’s disease. Psychol. Aging 25, 545–559. 10.1037/a0018498 20853964 PMC2946107

[B16] Johns E. K. Phillips N. A. Belleville S. Goupil D. Babins L. Kelner N.. (2012). The profile of executive functioning in amnestic mild cognitive impairment: disproportionate deficits in inhibitory control. J. Int. Neuropsych. Soc. 18, 541–555. 10.1017/s1355617712000069 22370245

[B17] Jonides J. Smith E. E. Koeppe R. A. Awh E. Minoshima S. Mintun M. A. (1993). Spatial working memory in humans as revealed by PET. Nature 363, 623–625. 10.1038/363623a0 8510752

[B18] Kane M. J. Engle R. W. (2002). The role of prefrontal cortex in working memory capacity, executive attention and general fluid intelligence: an individual-differences perspective. Psychon. Bull. Rev. 9, 637–671. 10.3758/bf03196323 12613671

[B19] Lee H. Baniqued P. L. Cosman J. Mullen S. McAuley E. Severson J. (2012). Examining cognitive function across the lifespan using a mobile application. Comput. Hum. Behav. 28, 1934–1946 10.1016/j.chb.2012.05.013

[B20] Luo L. Craik F. I. M. (2008). Aging and memory: a cognitive approach. Can. J. Psychiatry 53, 346–353. 18616854 10.1177/070674370805300603

[B21] Mayes A. R. Holdstock J. S. Isaac C. L. Montaldi D. Grigor J. Gummer A.. (2004). Associative recognition in a patient with selective hippocampal lesions and relatively normal item recognition. Hippocampus 14, 763–784. 10.1002/hipo.10211 15318334

[B22] Old S. R. Naveh-Benjamin M. (2008). Differential effects of age on item and associative measures of memory: a meta-analysis. Psychol. Aging 23, 104–118. 10.1037/0882-7974.23.1.104 18361660

[B23] Owen A. M. Downes J. J. Sahakian B. J. Polkey C. E. Robbins T. W. (1990). Planning and spatial working memory following frontal lobe lesions in man. Neuropsychologia 28, 1021–1034. 10.1016/0028-3932(90)90137-d 2267054

[B24] Passingham R. E. (1985). Memory of monkeys ( *Macaca mulatta*) with lesions in prefrontal cortex. Behav. Neurosci. 99, 3–21. 10.1037/0735-7044.99.1.3 4041231

[B25] Raz N. (2000). “Aging of the brain and its impact on cognitive performance: integration of structural and functional findings,” in The Handbook of Aging and Cognition, eds Craik F. I. M. Salthouse T. (Hillsdale, NJ: Erlbaum), 1–91.

[B26] Raz N. Rodrigue K. M. (2006). Differential aging of the brain: patterns, cognitive correlates and modifiers. Neurosci. Biobehav. R. 30, 730–748. 10.1016/j.neubiorev.2006.07.001 16919333 PMC6601348

[B27] Rosenbaum R. S. Murphy K. J. Rich J. B. (2012). The amnesias. Cognitive Sci. 3, 47–63 10.1002/wcs.155 26302472

[B28] Salthouse T. A. (2010). Selective review of cognitive aging. J. Int. Neuropsych. Soc. 16, 754–760. 10.1017/s1355617710000706 20673381 PMC3637655

[B29] Salthouse T. A. Meinz E. J. (1995). Aging, inhibition, working memory and speed. J. Gerontol. Psychol. 50B, P297–P306. 10.1093/geronb/50b.6.p297 7583809

[B30] Salthouse T. A. Toth J. Daniels K. Parks C. Pak R. Wolbrette M.. (2000). Effects of aging on the efficiency of task switching in a variant of the trail making test. Neuropsychology 14, 102–111. 10.1037//0894-4105.14.1.102 10674802

[B31] Sánchez-Cubillo I. Periáñez J. A. Adrover-Roig D. Rodríguez-Sanchez J. M. Ráos-Lago M. Tirapu J.. (2009). Construct validity of the trail making test: role of task-switching, working memory, inhibition/interference control and visuomotor abilities. J. Int. Neuropsychol. Soc. 15, 438–450. 10.1017/s1355617709090626 19402930

[B32] Scharre D. W. Chang S. I. Nagaraja H. N. Yager-Schweller J. Murden R. A. (2014). Community cognitive screening using the Self-Administered Gerocognitive Examination (SAGE). J. Neuropsych. Clin. N. 24, 64–71 10.1176/appi.neuropsych.13060145 24419587

[B33] Social Security Administration (2013). Top Names Over The Last 100 Years. Available online at: www.ssa.gov/OACT/babynames/decades/century.html.

[B34] Statistics Canada (2012a). Age Characteristics for Canada, 2011 Census, 100% Data. Available online at: www12.statcan.gc.ca/census-recensement/2011/dp-pd/prof/index.cfm?Lang=E.

[B35] Statistics Canada (2012b). Population 15 Years and Over by Highest Certificate, Diploma or Degree, by Age Groups, 2006 Census. Available online at: www.statcan.gc.ca/tables-tableaux/sum-som/l01/cst01/educ43a-eng.htm.

[B36] Stroop J. R. (1935). Studies of interference in serial verbal reaction. J. Exp. Psychol. 18, 643–662 10.1037/h0054651

[B37] Stuss D. T. Bisschop S. M. Alexander M. P. Levine B. Katz D. Izukawa D. (2001a). The trail making test: a study in focal lesion patients. Psychol. Assess. 13, 230–239. 10.1037/1040-3590.13.2.230 11433797

[B38] Stuss D. T. Floden D. Alexander M. P. Levine B. Katz D. (2001b). Stroop performance in focal lesion patients: dissociation of processes and frontal lobe lesion location. Neuropsychologia 39, 771–786. 10.1016/s0028-3932(01)00013-6 11369401

[B39] Tombaugh T. N. (2004). Trail making test A and B: normative data stratified by age and education. Arch. Clin. Neuropsych. 19, 203–214. 10.1016/s0887-6177(03)00039-8 15010086

[B40] Troyer A. K. D’Souza N. A. Vandermorris S. Murphy K. (2011). Age-related differences in associative memory depend on the types of associations that are formed. Neuropsychol. Dev. Cogn. B Aging Neuropsychol. Cogn. 18, 340–352. 10.1080/13825585.2011.553273 21390876

[B41] Troyer A. K. Murphy K. J. Anderson N. D. Craik F. I. M. Moscovitch M. Maione A.. (2012). Associative recognition in mild cognitive impairment: relationship to hippocampal volume and apolipoprotein E. Neuropsychologia 50, 3721–3728. 10.1016/j.neuropsychologia.2012.10.018 23103838

[B42] Troyer A. K. Murphy K. J. Anderson N. D. Hayman-Abello B. A. Craik F. I. M. Moscovitch M. (2008). Item and associative memory in amnestic mild cognitive impairment: performance on standardized memory tests. Neuropsychology 22, 10–16. 10.1037/0894-4105.22.1.10 18211151

[B43] United States Census Bureau (2011). The Older Population in the United States: 2011. Available online at: www.census.gov/population/age/data/2011.html.

[B44] Verhaeghen P. De Meersman L. (1998). Aging and the Stroop effect: a meta-analysis. Psychol. Aging 13, 120–126. 10.1037//0882-7974.13.1.120 9533194

[B45] Wagner N. Hassanein K. Head M. (2010). Computer use by older adults: a multi-disciplinary review. Comput. Human Behav. 26, 870–882 10.1016/j.chb.2010.03.029

[B46] Wechsler D. (1997). WAIS-III/WMS-III Technical Manual. San Antonio: Psychological Corporation.

[B47] Wechsler D. (2009). WMS-IV Technical and Interpretive Manual. San Antonio: Pearson.

[B48] Weintraub S. Dikmen S. S. Heaton R. K. Tulsky D. S. Zelazo P. D. Bauer P. J.. (2013). Cognition assessment using the NIH Toolbox. Neurology 80, S54–S64. 10.1212/WNL.0b013e3182872ded 23479546 PMC3662346

[B49] Welsh K. A. Breitner J. C. S. Magruder-Habib K. M. (1993). Detection of dementia in the elderly using telephone screening of cognitive status. Neuropsychiatry Behav. Neuropsychology 6, 103–110.

[B50] World Health Organization (2014). World Health Statistics 2014. Geneva: WHO Press.

[B51] Zakzanis K. K. Azarbehi R. (2014). Introducing BRAINscreen: web-based real-time examination and interpretation of cognitive function. Appl. Neuropsychol. Adult 21, 77–86. 10.1080/09084282.2012.742994 24826500

[B52] Zakzanis K. K. Mraz R. Graham S. J. (2005). An fMRI study of the trail making test. Neuropsychologia 43, 1878–1886. 10.1016/j.neuropsychologia.2005.03.013 16168730

